# Perioperative risk assessment for successful kidney transplant in leigh syndrome: a case report

**DOI:** 10.1186/s12882-018-0816-6

**Published:** 2018-02-01

**Authors:** Kathryn Ducharlet, Dominic Thyagarajan, Francesco Ierino, Lawrence P. McMahon, Darren Lee

**Affiliations:** 1 Department of Renal Medicine, Eastern Health Clinical School, Monash University, Level 2, 5 Arnold Street, Box Hill, Clayton, VIC Australia; 2 Department of Neurosciences, Monash Health, 246 Clayton Road, Clayton, VIC Australia; 3 Department of Nephrology, St Vincent’s Hospital Melbourne, 55 Victoria Parade, Fitzroy, VIC Australia

**Keywords:** Leigh syndrome, End stage renal disease, Transplant, Mitochondrial disorder, Dialysis

## Abstract

**Background:**

Leigh syndrome (LS) is a rare neurodegenerative mitochondrial disorder which typically presents in childhood but has a varied clinical course. Renal involvement such as proximal tubulopathy in patients with mitochondrial disorders has been described. However, end stage renal disease (ESRD) is uncommon and literature regarding patients undergoing kidney transplantation is limited. Successful deceased donor renal transplant has not been previously described in a patient with Leigh Syndrome.

**Case presentation:**

We report a 21-year-old Han Chinese man who presented with limb weakness and unsteady gait, which progressed rapidly over a period of months until he was wheelchair-bound. He subsequently developed ESRD and was commenced on hemodialysis. Investigations revealed a m.13513G > A mutation with clinical and radiological features consistent with LS. His mitochondrial disease stabilised and he underwent a multidisciplinary assessment for deceased donor kidney transplantation to identify and minimise the LS-associated perioperative risks and potential negative effects of immunosuppressants on his LS. Successful kidney transplantation followed with excellent graft function three and a half years post-transplant and improvement in the patient’s physical function.

**Conclusion:**

This case highlights the importance of careful pre-transplant perioperative risk assessment and post-transplant care in a rare and heterogeneous neurological disease to achieve an ultimately excellent clinical outcome. To our knowledge, this is the first report of successful deceased donor kidney transplant in a patient with known LS.

## Background

Leigh syndrome (LS) is a multisystem, rare neurodegenerative mitochondrial disorder that typically presents in children less than 1 year of age with an estimated incidence of 1 in 40,000 live births [1]. It is a clinically and genetically heterogeneous disease characterised by symmetrical necrotic lesions along the brainstem, diencephalon, and basal ganglia and more than 75 pathogenic genetic mutations identified [2]. Mutations in mitochondrial deoxyribonucleic acid (mtDNA) and nuclear DNA encoded subunits of respiratory chain complex 1 subunit genes have been reported, including the m.13513G > A mutation in the mitochondrially-encoded ND5 gene at low levels [3] which is associated with mitochondrial encephalomyopathy with lactic acidosis and stroke-like episodes (MELAS) or LS [4].

We report a man with late-onset LS associated with the m.13513G > A mutation complicated by ESRD, treated with hemodialysis followed by a deceased donor kidney transplant. In the literature, renal involvement in patients with mitochondrial disorders, most commonly tubular defects have been described [5]. Renal transplantation was considered in this case since his LS was not progressive. However, literature on kidney transplantation in patients with LS is limited given the often progressive and fatal nature of the disease in early childhood and the relatively rare occurrence of patients with conditions requiring transplantation. Solid organ transplantation in primary mitochondrial disease has been recently reviewed in a retrospective study of 35 patients from 17 Mitochondrial Disease Centers across North America, the United Kingdom and Australia [6]. This study highlights the need for further safety and efficacy studies on transplantation (particularly longitudinal ones) in primary mitochondrial disease. Firstly, post-transplant complications did not appear to be the same across all mitochondrial disease groups. Of the 9 patients with immediate post-transplant disease worsening, 3 of 9 had POLG gene mutations, who were the patients faring least well after transplantation. Secondly, of the 12 patients undergoing renal transplantation, only 9 had mtDNA point mutations and none of them had LS or the m.13513G > A mutation, which is a mutation known to have different phenotypic expressions of MELAS or LS, perhaps depending on mutant load [3, 4]. More literature is required so that we can better appreciate the variability of response to solid organ transplantation in these relatively rare but diverse patients.

This case highlights a considered approach for perioperative assessment and management for successful kidney transplantation in a patient with LS due to an uncommon mtDNA point mutation.

## Case report

A 21-year-old Han Chinese male finance officer first presented in China with weakness and unsteady gait, which progressed rapidly over months until he was wheelchair-bound. At age 23, he re-presented to our health service with seizures and ESRD requiring hemodialysis on a background of progressive weight loss, dysarthria and dysphagia. The cause of his ESRD was uncertain as a kidney biopsy was not performed prior to his migration to Australia.

His developmental history included a mild delay described in China at age 16; however, no cognitive impairment affecting his education or employment was noted. There was no family history of neurodegenerative or kidney disease. Physical examination showed a thin man (77 pounds and body mass index 14) with truncal imbalance, and generalised hypotonia, weakness and hyporeflexia.

To diagnose his neurodegenerative illness, investigations revealed normal serum lactate at 0.20 (0.05–0.24) mg/dL, however false negatives were possible as venous lactate is removed on hemodialysis. His cerebrospinal fluid analysis showed a normal pyruvate level of 0.12 (0.06–0.13) mmol/L, reduced glucose at 32.1 (50–80) mg/dL and raised protein at 78 (15–45) mg/dL. MRI brain showed severe cerebellar atrophy and milder atrophy in the brainstem and cerebral hemispheres and patchy increased T2 signal in the right cerebral hemisphere and the medulla. Mutations in mtDNA m.A3243G > A, m.8344A > G and m.8993 T > G were not identified in China and further genetic sequencing detected mtDNA mutation at m.13513G > A with 25% mutation load. The diagnosis of a mitochondrial disorder, most likely LS was made without a muscle biopsy.

Three years after commencing dialysis, his physical function improved modestly after a period of rehabilitation and home modifications. He remained seizure-free and suffered only occasional myoclonic jerks after cessation of carbamazepine with no ongoing anticonvulsant therapy. As his neurological state was non-progressive with a more favourable prognosis, the possibility of transplantation was entertained. Specific perioperative risks related to his LS and possible negative effects of immunosuppressants on his neurological disease were considered and addressed as part of his pre-transplant evaluation (Table 1).

**Table 1:** Potential Risks of Kidney Transplantation with Leigh Syndrome (LS)

Risks of immunosuppression worsening LS	Risks of transplantation worsened by LS
Mitochondrial function worsened by calcineurin inhibitors (CNIs) • *Trial of immunosuppresssants pre-transplant* Seizure threshold lowered by CNIs • *Trial of immunosuppressants pre-transplant*	Cardiac: • Severe left ventricular hypertrophy and diastolic dysfunction on echocardiogram • *Intensive care unit (ICU) monitoring to avoid “underfilling” (= > hypotension = > graft thrombosis) and “overfilling” (= > pulmonary oedema)* • *Deceased donor kidneys at risk of prolonged DGF avoided (AKI, DCD)* • *Holter monitor to exclude ventricular tachyarrhythmia; beta blocker use* Anesthesia: • Malignant hyperthermia and lactic acidosis ○ *Volatile agents and suxamethonium avoided; ICU monitoring* Low body weight • Excessive immunosuppression = > increased toxicity ○ *Reduced MMF starting dose (750 mg bd); further dose reduction according to therapeutic drug monitoring* Swallowing difficulty and uncertain oral absorption of immunosuppressants • Inadequate immunosuppression = > rejection and graft loss ○ *Trial of immunosuppressants and drug level measurements* Uncertain neurological prognosis • Risk of premature death with a functioning graft ○ Careful multidisciplinary approach to minimise LS-associated risks

The patient had asymptomatic but severe left ventricular hypertrophy with moderate diastolic dysfunction related to mitochondrial disease on transthoracic echocardiogram and ventricular tachyarrhythmia was not detected on Holter monitor. Offers of deceased donor kidneys with high risk of prolonged delayed graft function (donation after circulatory death and/or acute kidney injury) were declined to minimise cardiac risks early post-transplant.

An anesthetic review pre-transplant waitlisting was sought to evaluate the risk of cardiorespiratory and metabolic complications. The risks of potential metabolic disturbance including lactic acidosis, and malignant hyperthermia or rhabdomyolysis caused by volatile agents [7] were considered. Anesthetics, including volatile agents, propofol and thiopentone pose a theoretical risk as they act on tissues with high energy requirements and decrease mitochondrial function. However, reports of mitochondrial disease patients having anesthetics indicate that they may generally be used without adverse outcomes [8]. For example, in 122 patients with mitochondrial disease undergoing muscle biopsy with a mean operation time of 36 min, 83 of whom had measurable deficits of oxidative phosphorylation. However, there were no anesthetic complications following standard preoperative assessment, monitoring and induction [9]. Seventeen of these patients received propofol as an induction agent. Although mitochondrial disease has been proposed as a risk for the propofol infusion syndrome - acute bradycardia progressing to asystole, lipemic plasma, fatty liver enlargement, metabolic acidosis, rhabdomyolysis or myoglobinuria associated with prolonged high dose propofol infusions (> 4 mg/h for > 48 h) [10] this has not been proven [8]. Thus, we considered a short induction and limited propofol boluses would be safe for anesthesia in our patient. Malignant hyperthermia has not been proven to be a special risk in mitochondrial patients [8].

Calcineurin inhibitors may worsen neurological diseases by altering mitochondrial function [11, 12], myopathy from Fanconi syndrome [12, 13] and lowering seizure threshold [14]. Devastating reversible neurotoxicity secondary to calcineurin inhibitors has been reported post-transplant [15]. For our patient while on dialysis, a two-week trial of tacrolimus and mycophenolate caused no deterioration of mitochondrial disorder, and achieving adequate drug levels suggested his ability to swallow and absorb the immunosuppressants.

The patient’s low body weight was related to his neuromuscular disease, causing asthenia, early satiety and oropharyngeal fatigue. Optimising the quality of nutritional intake is known to improve mitochondrial health in patients with mitochondrial disorders [16] and nutrition was optimised with dietetic input, education and liquid oral supplementations.

The patient underwent a deceased brain dead donor kidney transplant at age 27 with 3/6 HLA mismatches and no DR mismatch. He weighed 75 pounds (34 kg) and had 150 mg of propofol as induction and another 50 mg in small boluses as maintenance (200 mg in total) during a relatively short anesthetic of 2 h and 15 min. Suxamethonium was avoided and there was no complication of malignant hyperthermia or lactic acidosis. He was managed in intensive care post-operatively for hemodynamic monitoring and fluid management without inotropic requirements, and he required hemodialysis twice for delayed graft function. Standard immunosuppression included basiliximab induction and maintenance therapy with prednisolone, tacrolimus, and mycophenolate mofetil at a reduced dose of 750 mg twice daily given his low body weight.

Four months post-transplant, he developed acute on chronic diarrhoea and associated dehydration. Histopathology from colonoscopy showed features of mild pancolitis without features of cytomegalovirus infection. This was considered to be mycophenolate-related and his dose was reduced to 500 mg twice daily with partial symptomatic improvement. Therapeutic drug monitoring with mycophenolic acid area under the curve (MPA AUC0-12 h) was measured and reported as 104 (target range 30–60) mg.h/L and further dose reduction to 500 mg mane 250 mg nocte resulted in resolution of symptoms.

Graft function has remained stable at three and a half years with a serum creatinine between 1.0 and 1.2 mg/dL and no proteinuria. Twelve-month protocol biopsy showed no features of rejection or fibrosis. His severe left ventricular hypertrophy remained unchanged on transthoracic echocardiogram 5, 21 and 40 months post-transplant, consistent with his mitochondrial disease.

The patient’s weight increased to 88 pounds and he can now independently feed and toilet himself and stand for longer periods. His dysphagia and dysarthria were however unchanged. The patient continues to live in the community with improved quality of life and freedom from dialysis and he has recently returned to Australia following a vacation to China.

## Discussion

Experience and literature regarding patients with mitochondrial disorders receiving a kidney transplant is limited. One case report described a 57-year-old man who developed ESRD at age 48, and MELAS was not diagnosed until 5 years post-transplant after a 40-year course of multisystem illness [17]. Non-English publications have reported successful living donor kidney transplant [18] and simultaneous pancreas and kidney transplant [19] in patients with mitochondrial disorders. Although a recent case series reported 12 patients with mitochondrial disorders undergoing kidney transplantation [6], to our knowledge, this is the first report of a patient with LS receiving a successful deceased donor kidney transplant.

Considerations regarding the risk and benefit of kidney transplantation compared with the poor survival and morbidity of ongoing hemodialysis were complex in this patient. National guidelines in Australia and New Zealand recommend that candidates for the deceased donor waiting list should have low perioperative mortality risk and greater than 80% 5-year post-transplant survival due to the scarcity of deceased donor organs and need to optimise resources by minimising the risk of premature patient death with a functioning transplant [20]. Similar guidelines also exist internationally [21]. However, transplantation improves quality of life and long-term survival compared with maintenance dialysis [22]. No living donor candidates were available, which would have reduced perioperative risk compared with a deceased donor kidney transplant [23] and avoided the risk of suboptimal utility of deceased donor organs in a patient with an uncertain prognosis.

The patient experienced a modest improvement in physical function and independence three and a half years post-transplant. The effect of uremia on mitochrondrial function is unknown. Given the usually progressive nature of LS, restoration of kidney function and freedom from hemodialysis may have contributed to this unexpected improvement.

LS is a rare neurodegenerative disease that typically presents in childhood and is usually progressive and lethal by early adulthood. However, age of onset, neurological severity and prognosis can vary, as evidenced by our case. There is limited literature regarding patients with LS, ESRD and kidney transplantation. This case highlights the importance of careful pre-transplant perioperative risk assessment and post-transplant management to achieve an excellent outcome, and these patients should be considered for kidney transplantation.

## Abbreviations

ESRD: End stage kidney disease
LS: Leigh syndrome
MELAS: Mitochondrial encephalomyopathy with lactic acidosis and stroke-like episodes
mtDNA: Mitochondrial deoxyribonucleic acid
